# A successful combined spinal-epidural anesthesia for cesarean section in a patient with neurofibromatosis type 1-associated dural ectasia

**DOI:** 10.1186/s40981-024-00745-w

**Published:** 2024-10-01

**Authors:** Hitomi Taka, Nobuyoshi Kusama, Minami Sakamoto, Nobuko Sasano, Motoshi Tanaka

**Affiliations:** 1 Department of Anesthesiology and Intensive Care Medicine, Nagoya City University West Medical Center, 1-1-1, Hirate-Cho, Kita-Ku, Nagoya, Aichi 462-8508 Japan; 2 Department of Anesthesiology and Intensive Care Medicine, Nagoya City University Graduate School of Medical Sciences, 1, Kawasumi, Mizuho-cho, Mizuho-ku, Nagoya, Aichi 467-8601 Japan

**Keywords:** Neurofibromatosis type 1, Dural ectasia, Failed spinal anesthesia

## Abstract

**Background:**

Dural ectasia is a common manifestation of neurofibromatosis type 1. Although there have been reports of unsuccessful spinal anesthesia due to dual ectasia in Marfan syndrome, reports describing similar unsuccessful spinal anesthesia in neurofibromatosis type 1 are lacking.

**Case presentation:**

A parturient with neurofibromatosis type 1 was scheduled for a repeat cesarean section. During a previous cesarean section, she had experienced a failed spinal anesthesia, which resulted in a conversion to general anesthesia. Preoperative lumbar magnetic resonance imaging revealed dural ectasia, which was speculated to be the cause of the previous spinal anesthesia failure. Therefore, combined spinal-epidural anesthesia was implemented. Because the block level of spinal anesthesia was insufficient as predicted, supplemental administration of epidural anesthesia successfully provided adequate analgesia for the surgery.

**Conclusions:**

Combined spinal-epidural anesthesia can be useful for the management of cesarean sections in patients with neurofibromatosis type 1-associated dural ectasia.

## Background

Neurofibromatosis type 1 (NF-1) is an autosomal dominant disorder that affects the ectodermal and mesodermal tissues [1]. NF-1 patients present various clinical manifestations, including in the spine and nervous system [1, 2]. The presence of scoliosis or spinal cord tumors renders the use of neuraxial anesthesia difficult [1]. Dural ectasia, defined as ballooning or widening of the dural sac, is one of the common neuraxial manifestations of NF-1 [2, 3]. While the association between dural ectasia in patients with Marfan syndrome and spinal anesthesia failure is well documented [4, 5], similar occurrences in NF-1 have not been reported. We herein report a case of cesarean section (C-section) in an NF-1 patient with dural ectasia who had a history of failed spinal anesthesia during the previous C-section.

## Case presentation

A 34-year-old parturient (158 cm, 49.8 kg, gravida 2, para 1) was referred and admitted to our hospital for severe preeclampsia at 24 weeks’ gestation. During childhood, she was diagnosed with NF-1 but did not receive any follow-up care. During her previous pregnancy at 31 years old, she underwent a C-section at 38 weeks’ gestation due to a breech presentation at a different medical facility, where spinal anesthesia was converted to general anesthesia due to an inadequate block. At 32 years old, she underwent right total hip replacement for osteoarthritis under general anesthesia at a different medical facility.

**Fig. 1:**
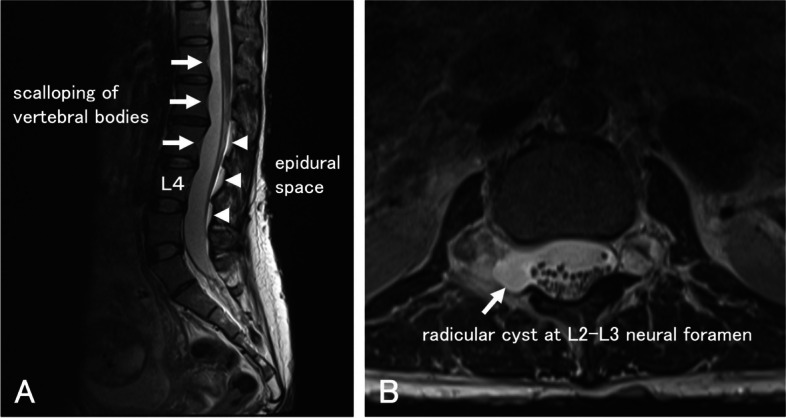
**a** Sagittal MRI showed widening of the dural sac and posterior scalloping of the vertebral bodies from L1 to L3 (arrows). Posterior segment of the epidural space was observed (arrow heads). **b** Axial MRI at L2-3 showed a radicular cyst expanding the right neural foramen (arrow)

In anticipation of a repeat C-section, she was referred for a preanesthesia consultation at 25 weeks’ gestation. Her blood pressure was controlled within the goal range with oral nifedipine. Blood and urine tests ruled out pheochromocytoma, a possible NF-1 complication. She had cutaneous neurofibromas and café-au-lait spots on the trunk. Neurological examination was normal, and no sensory or motor deficit was observed. Ultrasound examination revealed no subcutaneous masses that would interfere with the neuraxial anesthesia procedures. The distance from the skin to the posterior complex at the L3-4 level was 3 cm. We performed magnetic resonance imaging (MRI) to explore the possibility of spinal tumors. The MRI images showed no spinal tumors but revealed dilatation of the dural sac, posterior scalloping of the vertebral bodies, and a radicular cyst at the right L2-3 neural foramen (Fig. 1), which led to a diagnosis of dural ectasia. The dural ectasia, which may not have been previously detected, was suspected to be the cause of failed spinal anesthesia during the previous C-section. MRI imaging revealed an epidural space for possible epidural catheterization. The patient was informed of the risk–benefit of general versus neuraxial anesthesia, including the potential neurological complications associated with neuraxial anesthesia. After thoroughly explaining, we respected the patient’s preference to proceed with combined spinal-epidural anesthesia (CSEA) using the needle-through-needle technique.

An elective C-section was performed at 28 weeks’ gestation because of intrauterine fetal growth restriction. Local anesthesia with 1% lidocaine was administered under standard noninvasive monitoring and in the sitting position. An 18-gauge Tuohy needle (Smiths Medical Japan Ltd., Tokyo, Japan) was inserted into the epidural space at the L3–4 interspace. Subsequently, dural puncture was performed using a 27-gauge spinal needle (Smiths Medical Japan Ltd.). After checking aspiration for clear cerebrospinal fluid, 2.4 mL of 0.5% hyperbaric bupivacaine combined with 15 µg of fentanyl was intrathecally administered. Finally, an epidural catheter was placed 4 cm into the epidural space. As the bilateral sensory block level evaluated via a cold sensation was below L1 after 5 min, a total of 12 mL of 2% lidocaine with 1:200,000 epinephrine was administered in 3-mL increments through the epidural catheter. After confirming sensory block of the T6 level, the surgery was commenced. The patient delivered a 597-g infant with Apgar scores of 6 and 8 at 1 and 5 min, respectively. At the end of the surgery, the sensory block level was T4, and then the epidural catheter was removed before leaving the operating room. The postoperative course was uneventful without any complications, including postdural puncture headache, and the patient was discharged on postoperative day 7.

## Discussion

We experienced a successful CSEA for a C-section of a patient with NF-1-associated dual ectasis. When performing neuraxial anesthesia in patients with NF-1, dural ectasia should be considered as a potential complication. CSEA can be a useful anesthetic option.

To the best of our knowledge, this is the first case report of spinal anesthesia failure in an NF-1 patient due to dural ectasia. Dural ectasia has been extensively described in Marfan syndrome, with its prevalence ranging from 63 to 92% [6, 7]. A previous review of NF-1 found a 25.7% incidence of dural ectasia [2], suggesting that it is not a rare manifestation of NF-1. In cases of dural ectasia, spinal anesthesia failure is considered to be caused by increased cerebrospinal fluid volume in the distended dural sac, which restricts the spread of intrathecally injected local anesthetic [4, 5]. The success of spinal anesthesia differs based on the extent of dural ectasia [8]. Based on the grading system developed by Fattori et al. [7], our patient, the MRI of whom revealed expanded dural sac and small radicular cyst, was classified as having mild dural ectasia. The present case showed that even the presence of mild dural ectasia does not guarantee the success of spinal anesthesia.

Case reports describing CSEA in patients with Marfan syndrome having dural ectasia are scarce [5, 8, 9]. These reports indicated that while spinal anesthesia failed to provide adequate block levels for elective C-sections, epidural anesthesia was successfully employed without complications. Epidural anesthesia is not an absolute contraindication in patients with dural ectasia; however, it requires careful consideration and skillful technique due to the increased risk of dural puncture in moderate-to-severe cases [8]. In our case, the MRI images facilitated the selection of CSEA.

In an NF-1 parturient, the necessity of neuraxial imaging before neuraxial anesthesia is controversial. During pregnancy, neurofibromas can increase in size [10], and the possibility of spinal tumor growth is a potential risk of neuraxial anesthesia. Older publications recommended avoiding neuraxial anesthesia in the absence of neuraxial imaging [11, 12]. Meanwhile, the practice guideline from the American College of Medical Genetics and Genomics states that preanesthetic neuraxial imaging is probably not required [13]. However, these recommendations do not mention dural ectasia. Patients with dural ectasia may present with low back pain, radicular pain in the buttocks or legs, or headache, but most, like our case, are asymptomatic [14]. The lack of neuraxial imaging may lead to oversight of asymptomatic dural ectasia. Therefore, we consider that neuraxial imaging is beneficial to enhancing patient safety when performing neuraxial anesthesia in NF-1 patients. In a patient with increased intracranial pressure caused by an enlarged intracranial tumor, neuraxial anesthesia can result in brain herniation [11]. In the present case, we did not perform brain MRI due to the absence of symptoms, such as headache, nausea, vomiting, and visual disturbances. If there are concerns, a brain MRI may be advisable for safety assurance.

## Data Availability

Not applicable.

## Abbreviations

NF-1: Neurofibromatosis type1
C-section: Cesarean section
CSEA: Combined spinal-epidural anesthesia
MRI: Magnetic resonance imaging
